# Litchi seed extract inhibits epidermal growth factor receptor signaling and growth of Two Non-small cell lung carcinoma cells

**DOI:** 10.1186/s12906-016-1541-y

**Published:** 2017-01-05

**Authors:** Yuan-Chiang Chung, Chin-Hui Chen, Yu-Ting Tsai, Chih-Cheng Lin, Jyh-Ching Chou, Ting-Yu Kao, Chiu-Chen Huang, Chi-Hsuan Cheng, Chih-Ping Hsu

**Affiliations:** 1 Department of Surgery, Cheng-Ching Hospital, Chung-Kang Branch, Taichung, Taiwan; 2 Department of Health and Leisure Management, Yuanpei University of Medical Technology, Hsinchu, Taiwan; 3 Department of Medical Laboratory Detection, Lotung Poh-Ai Hospital, Yilan, Taiwan; 4 Department of Biotechnology and Pharmaceutical Technology, Yuanpei University of Medical Technology, Hsinchu, Taiwan; 5 Department of Natural Resources and Environmental Studies, National Dong Hwa University, Hualien, Taiwan; 6 Department of Medical Laboratory Science and Biotechnology, Yuanpei University of Medical Technology, No. 306 Yuanpei Street, Hsinchu, 30015 Taiwan; 7 Veterinary Medical Teaching Hospital of National Chung Hsing University, Taichung, Taiwan; 8 Department of Laboratory Medicine, Taipei Medical University Hospital, Taipei, Taiwan; 9 Department of Medicinal Botanicals and Health Applications, Da-Yeh University, Changhua, Taiwan

**Keywords:** Litchi seed extract, NSCLC, Cell cycle, Apoptosis, EGFR

## Abstract

**Background:**

Litchi seeds possess rich amounts of phenolics and have been shown to inhibit proliferation of several types of cancer cells. However, the suppression of EGFR signaling in non-small cell lung cancer (NSCLC) by litchi seed extract (LCSE) has not been fully understood.

**Methods:**

In this study, the effects of LCSE on EGFR signaling, cell proliferation, the cell cycle and apoptosis in A549 adenocarcinoma cells and NCI- H661 large-cell carcinoma cells were examined.

**Results:**

The results demonstrated that LCSE potently reduced the number of cancer cells and induced growth inhibition, cell-cycle arrest in the G1 or G2/M phase, and apoptotic death in the cellular experiment. Only low cytotoxicity effect was noted in normal lung MRC-5 cells. LCSE also suppressed cyclins and Bcl-2 and elevated Kip1/p27, Bax and caspase 8, 9 and 3 activities, which are closely associated with the downregulation of EGFR and its downstream Akt and Erk-1/-2 signaling.

**Conclusion:**

The results implied that LCSE suppressed EGFR signaling and inhibited NSCLC cell growth. This study provided *in vitro* evidence that LCSE could serve as a potential agent for the adjuvant treatment of NSCLC.

## Background

Lung cancer is the most threatening type of cancer in industrial countries, with high morbidity and mortality, because many patients are diagnosed with advanced disease [1]. Non-small cell lung cancer (NSCLC) is the most prevalent type of lung malignancy. Although recent advancements in treatment strategies using tyrosine kinase inhibitors (TKIs) to treat NSCLC have been developed to prolong patient survival by 3-fold as compared to traditional chemotherapies, drug resistance still occurs in a proportion of patients. Other adjuvant strategies to prolong the survival rate of NSCLC patients administered TKIs should be developed [2–4]. Litchi (*Litchi chinensis*, Sapindaceae) is a warm-climate fruit tree that originates from southern China and is cultivated in semi-tropical areas of the world for its delicious fruit [5]. In traditional Chinese herbal medicine, the function of litchi seeds are the release of stagnant humor and remote chilling, and they are used as analgesic agent capable of relieving cough, gastralgia, neuralgia, orchitis, colic, testicular swelling and smallpox [6]. In India, the seeds are powdered and administered to people with intestinal trouble. The seeds also have the reputation of relieving neuralgic pain in China. In recent studies, a variety of proanthocyanidins, flavonoid glycosides and polysaccharides were identified from litchi seeds [7–9]. Some of these compounds appeared to exhibit anti-neoplastic activities in breast cancer, sarcoma, nasopharynx cancer, lung cancer, cervical cancer and hepatocellular carcinoma cell lines [7, 8, 10, 11]. A possible mechanism may be due to the inhibition of EGFR signaling and concomitant induction of intrinsic and extrinsic apoptosis pathways [10]. The hypothesis of the present study is the potential application of litchi seed extract (LCSE) as an anti-lung cancer agent because of the ability to target EGFR. However, the detailed cellular and molecular mechanisms of the suppressive effect of LCSE in NSCLC cells are still unclear. In this study, we investigated the effects of LCSE on two NSCLC cell lines, adenocarcinoma A549 and large-cell carcinoma NCI-H661 cells, to evaluate the potential of LCSE for the treatment of NSCLC.

## Methods

### Materials

Chemicals used for the preparation of buffers, cell-staining fluorescent dyes or drugs such as proteinase inhibitor cocktail, sodium orthovanadate, sodium fluoride, sodium pyrophosphate, Triton X-100, ammonia persulfate, rhodamine 123, propidium iodide, *N,N,N’,N’*-tetramethylethylenediamine, sodium dodecyl sulfate (SDS), Tween 20, gallic acid and catechin were obtained from Sigma (St. Louis, MO, USA). Cell-culture materials such as Roswell Park Memorial Institute (RPMI) media 1640, Dulbecco’s Modified Eagle Medium (DMEM), fetal bovine serum (FBS), L-glutamine, trypsin and antibiotics were purchased from Gibco Ltd. (Paisley, UK). The protein assay kit (bicinchoninic acid; BCA) was purchased from Pierce (Rockford, IL, USA). Acrylamide was obtained from Bio-Rad (Hercules, CA, USA). Polyvinylidene fluoride (PVDF) membrane (Immobilon-P) was obtained from Millipore (Bedford, MA, USA). Mouse monoclonal anti-caspase 3, B cell lymphoma 2 (Bcl-2), cyclin B1, cyclin D1, cyclin E and anti-MAP kinase (Erk-1/-2) antibodies and rabbit anti-phosphor-Erk-1/-2 (The202/Tyr204) were purchased from Zymed (San Francisco, CA, USA). Rabbit polyclonal anti-human pan Akt, anti-phosphor-Akt (S473), anti-EGFR, anti-phosphor-EGFR (phospho Tyr1092) and anti-Kip1/p27, and goat polyclonal anti-pro-caspase 8, pro-caspase 9, poly [ADP-ribose] polymerase (PARP), Bcl-2 associated protein X (Bax) antibodies and goat anti-rabbit, anti-mouse and rabbit anti-goat secondary antibodies conjugated with horseradish peroxidase (HRP) were obtained from R&D Systems (Minneapolis, MN, USA). Annexin V conjugated with fluorescein isothiocyanate (FITC) was obtained from Gene Research (Taipei, Taiwan).

### Litchi seed extract

Hei Yeh Litchi (*Litchi* chinensis Sonn. var. Hei Yeh) fruit were purchased from Rayfoung Co., Ltd (Chiayi, Taiwn) and identified by Dr. Chih-Cheng Lin and Chih-Ping Hsu using the Digital Fruit Genetic of Taiwan database of the Agricultural Research Institute (Council of Agriculture, Executive Yuan of Taiwan) as a reference (https://kmweb.coa.gov.tw/subject/ct.asp?xItem=176011&ctNode=5525&mp=1&kpi=0&hashid=). Litchi seed extract was obtained using the method described in a previous report [12]. Briefly, litchi seeds dried in a 70 °C oven were ground using a stainless-steel grinder (RT-02, Rong Tsong Iron Factory Incorporation, Taiwan). Crude extract of litchi seeds was obtained by mixing the powder with 70% ethanol and refluxing overnight. The solution was then filtered and centrifuged to remove any undissolved materials. The supernatant was subsequently concentrated until no ethanol remained using a rotary evaporator under reduced pressure and a water bath <35 °C, which was then freeze-dried. The final crude extract was defined as LCSE. The total levels of phenols, flavonoids and condensed tannins were estimated using colorimetric methods as described previously [12].

### Cell culture

A549 and NCI-H661 cells were purchased from the Bioresource Collection and Research Center in Taiwan. A549 cells were established from lung carcinomatous tissue from a 58-year-old Caucasian male, and the cell type was identified as lung carcinoma. NCI-H661 cells were derived from the lymph node of a patient with large-cell lung cancer. These two cell lines were cultured in 90% RPMI 1640 with 2 g/L sodium bicarbonate, 10% heat-inactivated FBS, 25 U/mL penicillin and 25 μg/mL streptomycin. The cells were incubated at 37 °C in a 95% air/5% CO_2_ water-saturated atmosphere. All experiments were carried out using cell lines passaged between 5 and 20 times.

### Cell proliferation assay

Cells were plated at 100,000 cells per 60-mm tissue culture dish and then treated with LCSE (0, 12.5, 25, 50, 100, or 150 μg/mL) after approximately 18 h, when the cells had become attached to the bottom of the plates. Cells were incubated with LCSE for 24 h and then collected by trypsinization, stained with trypan blue, and counted in suspension in duplicate using a hemocytometer. Data were obtained from the averages of three independent experiments.

### Clonogenic growth assay

200 cells were seeded in a 6-well plate and treated with LCSE (1 ~ 50 μg/mL) then incubated at 37 °C for 14 days. On day 14, the colonies were fixed in 70% ethanol and stained with 0.2% crystal violet. Colonies of >50 cells were counted and the colony-forming potential of LCSE-treated NSCLC cells was expressed as a percentage of colonies of the untreated cells.

### Cell-cycle analysis

As described in a previous report [13], LCSE-treated cells were collected by trypsinization and then fixed in 70% ethanol at −20 °C for at least 30 min. Fixed cells were reconstituted in phosphate-buffered saline and then stained with propidium iodide solution (20 μg/mL propidium iodide and 10 μg/mL RNase A) at 37 °C in the dark for 30 min. The cell cycle of LCSE-treated cells was examined by flow cytometry (Becton Dickinson, CA) using FL-2A to score the DNA content of cells. The numbers of cells in the G1, S and G2/M cell-cycle phases were determined using Modfit software and expressed as the percentage of total cells (Verity Software House, Inc., Topsham, ME, USA).

### Apoptosis

Apoptosis of LCSE-treated cells was analyzed using annexin V-FITC labeling followed by flow cytometry, as described in previous reports [14]. The treated cells were trypsinized and suspended in binding buffer (10 mM HEPES, pH 7.4, 140 mM NaCl, and 2.5 mM CaCl_2_). Cells were stained with 2 μg/mL annexin V-FITC at room temperature in the dark for 30 min. The fluorescence intensity of the labeled cells was measured using FITC and flow cytometry with FL-1H. Untreated cells served as the negative control.

### Mitochondrial membrane potential *(*Δψ*m)*

***Δψ***m, the mitochondrial membrane potential, indicates the mitochondria membrane integrity in cells, and was assessed as described by Hsu *et al*. [15]. Briefly, LCSE-treated cells were collected, suspended at a density of 1 × 10^6^ cells/mL in fresh medium and stained with 10 *μ*g/mL rhodamine 123 for 30 min at 37 °C. Cells were then washed twice with fresh medium, and the fluorescence intensity of the cells was immediately examined by flow cytometry using FL-1H. Ten-thousand cells without cell debris were analyzed, and the rhodamine 123-negative cells were defined as those with a lower fluorescence intensity than the untreated cells.

### Immunoblotting

Ice-cold phosphate-buffered saline-washed cells were lysed in homogenization buffer (10 mM Tris-HCl at pH 7.4, 2 mM EDTA, 1 mM EGTA, 50 mM NaCl, 1% Triton X-100, 50 mM NaF, 20 mM sodium pyrophosphate, 1 mM sodium orthovanadate, and 1:100/v:v proteinase inhibitor cocktail) at 4 °C for 30 min. Cell extracts were obtained by ultracentrifugation of cell lysates at 100,000 × *g* for 30 min at 4 °C. The protein concentration of the cell extract was measured using a protein assay kit, and the extracts were adjusted to 2 mg/mL with homogenization buffer. For immunoblotting analysis, the proteins in the cell extracts were separated by SDS-PAGE and electrotransferred to PVDF membranes using semi-dry blot apparatus (Bio-Rad) at 3 mA per cm^2^ of gel in transfer buffer (25 mM Tris, pH 8.3, 192 mM glycine, and 20% methanol) at room temperature for 30 min. The free protein-binding sites on the PVDF membrane were saturated by incubation with 5% nonfat milk in Tris-buffered saline with Tween 20 (TTBS) (20 mM Tris at pH 7.4, 0.15 M NaCl, and 0.2% Tween 20) at 25 °C for 2 h. The membrane was then incubated with 0.1 μg/ml primary antibody in TTBS containing 3% nonfat milk at 4 °C overnight and subsequently with secondary antibody conjugated with peroxidase (1:1000) at 25 °C for 1 h. The immunoblots were developed using an enhanced chemiluminescence system, and the luminescent images were captured using a Chemiluminescent Detection System (Chemi Doc. XRS, Bio-Rad).

### Statistical analysis

All data are expressed as means ± SD unless stated otherwise. The differences between groups were calculated using Student’s unpaired *t*-test. Dose-dependent effects were calculated using simple linear regression. *P* < 0.05 was regarded as statistically significant. All statistical analyses were performed using SPSS version 17.0 (SPSS, Inc., Chicago, IL, USA).

## Results

### The inhibitory effect of LCSE on the growth of NSCLC cell lines

**Fig. 1:**
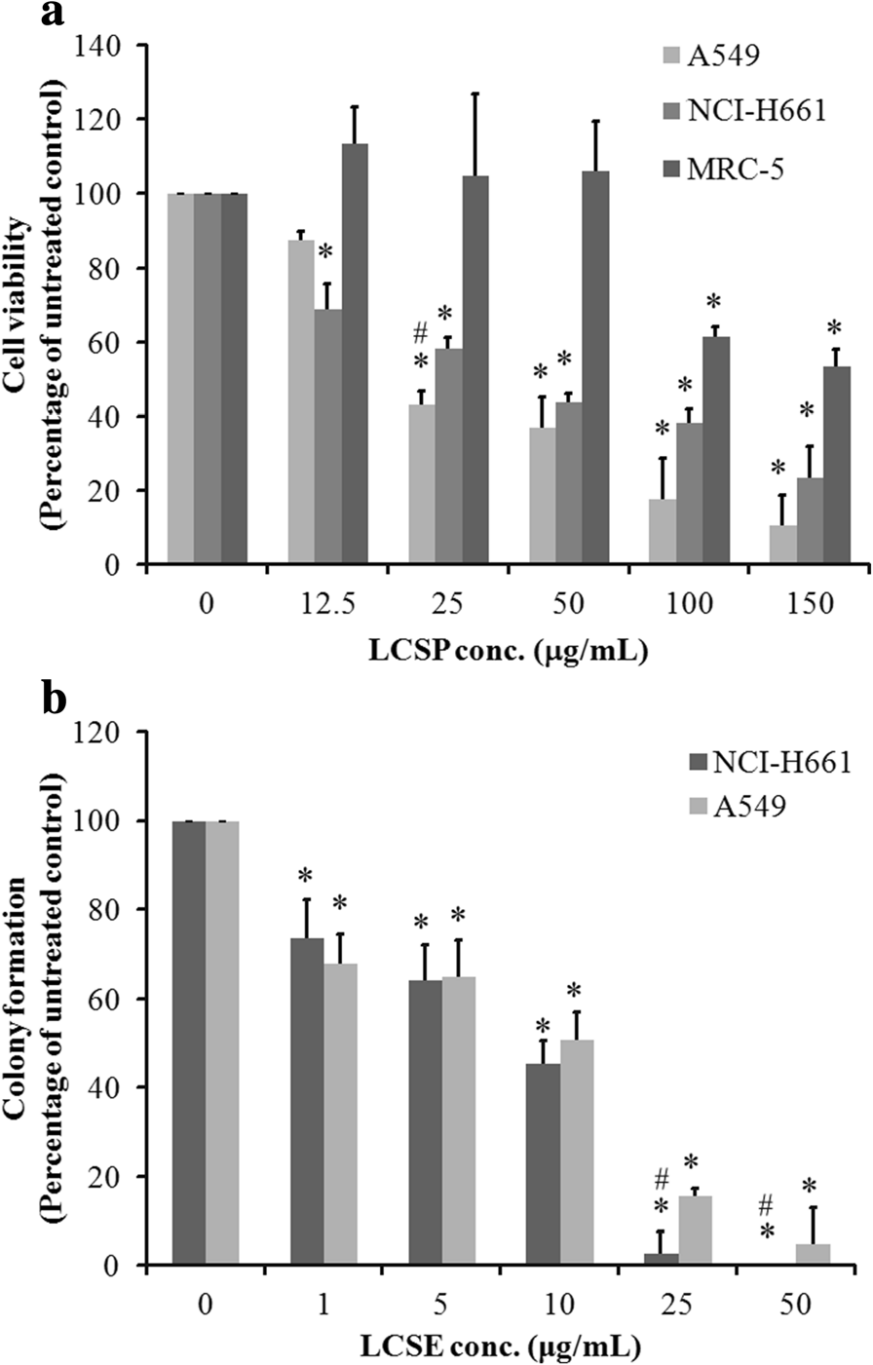
Dose-dependent responses of A549, NCI-H661 and MRC-5 cells to LCSE. Cells were treated with increasing concentrations of LCSE as indicated and then incubated at 37 °C for 24 h to obtain a dose-dependent response. Viable cells were stained with trypan blue and counted under a microscope. Cell viability was expressed as the percentage of untreated cells. **a**. Colony formation of LCSE-treated cells was assessed by seeding 200 cells in a 6-well plate and treating with LCSE (1 ~ 50 μg/mL), followed by incubation at 37 °C for 14 days. On day 14, the colonies were fixed in 70% ethanol and stained with 0.2% crystal violet. Colonies of >50 cells were counted and the colony-forming potential of LCSE-treated NSCLC cells was expressed as the percentage of colonies of the untreated cells (**b**). Data are the average of three independent experiments and are expressed as means ± SD. *P < 0.05 (compared with untreated control); # *P* <0.01 (compared within A549 and NCI-H661)

The proliferation and colony-forming activities of A549 and NCI-H661 cells were both inhibited by LSCE treatment. As shown in Fig. 1a, the cell survival ratio decreased gradually in both treated cell lines as compared to the untreated cells after 24 h. The sensitivity of the A549 cell line to LCSE was greater than that of NCI-H661 when the dose of LCSE was higher than 25 μg/mL. MRC-5, a human normal lung cell line, was not remarkably influenced by treatment with LCSE at dosage lower than 50 μg/mL. In cancer cell lines, LCSE exhibited approximately 60% growth inhibition with dosage of 100 μg/mL LCSE, while the survival rate decreased to 20% in A549 and 30% in NCI-H661 cells after treatment with 150 μg/mL of LCSE. Growth inhibition of NSCLC cells caused by LCSE treatment was similarly observed in colony-forming activity. As shown in Fig. 1b, the number of colonies gradually reduced as the dosage of LCSE increased. The sensitivities of the two cell lines to LCSE in this assay had similar trend; however, NCI-H661 cells exhibited greater sensitivity than A549 cells in the dosage of 25 μg/mL and 50 μg/mL (*P* < 0.01). These results demonstrated that LCSE exerted significant inhibition on both the proliferation and colony formation in these two NSCLC cell lines, whereas the cytotoxicity effects in normal lung cell line were relatively low.

### Cell-cycle arrest at the G1 or G2/M phase by LCSE treatment

**Fig. 2:**
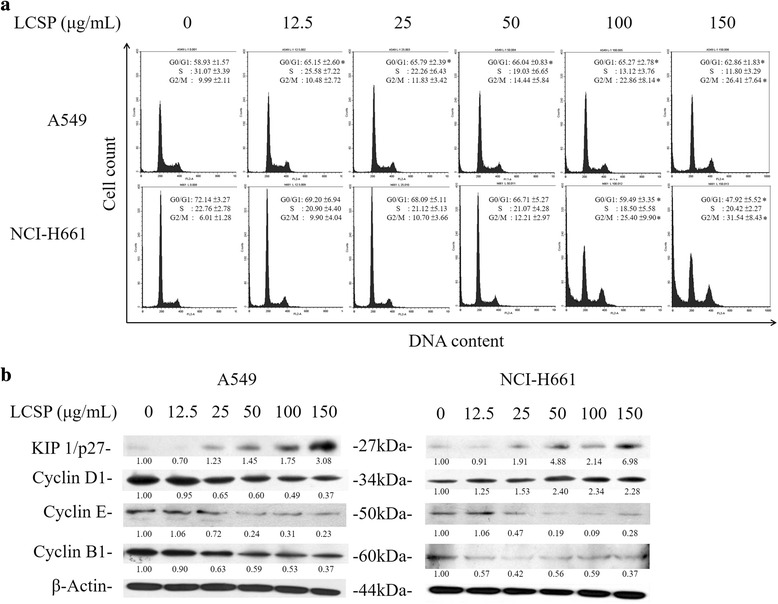
Cell-cycle arrest in NSCLC cells by LCSE. LCSE-treated cells were incubated at 37 °C for 24 h, fixed in 70% alcohol and stained with propidium iodide, then analyzed by flow cytometry, as described in Materials and Methods. The same LCSE-treated cells were lysed and the cell proteins separated by SDS-PAGE followed by immunoblotting to show proteins as indicated, with the beta-actin level as the loading control. Protein levels were quantified using Image Lab software (Bio-Rad) according to the density of each band on the immunoblotting image, normalized to the reference band (β-actin) and presented as the fold change of the untreated control. The cell-cycle distributions of LCSE-treated A549 and NCI-H661 cells are shown in (**a**), respectively, and the cell-cycle-associated proteins are shown in (**b**). The data reported are the percentages of total cells from the averages of three independent experiments and are expressed as means ± SD. *P < 0.05

The cell-cycle distribution of LCSE-treated NSCLC cells is shown in Fig. 2a. A549 cells exhibited increasing percentage of G1-phase cells upon LSCE treatment with dosage greater than 12.5 μg/mL. The percentage of total control cells increased from 58.93 to 66.04% as the dosage elevated from 12.5 μg/mL to 50 μg/mL of LCSE. Increasing numbers of cells of G2/M-phase were also noted, from 9.99% of the total control cells to 26.41% in the group of 150 μg/mL LCSE-treated cells. Regarding to the number of S-phase, LCSE-treated cells gradually decreased from 31.07% of the total control cells of 12.5 μg/mL to 11.8% of 150 μg/mL in LCSE-treated cells. NCI-H661 cells also showed a trend of gradual increase in G2/M-phase cells, from 6.01% of the total control cells to 31.54% of 150 μg/mL LCSE-treated cells, but the numbers of cells in G1-phase were reduced. The changes in cell-cycle controlling proteins are shown in Fig. 2b. The levels of cell-cycle controlling proteins, including cyclin D1, E, and B1, decreased gradually, and the level of Kip1/p27 increased after LCSE treatment in A549 cells. The levels of cyclin E and B1 decreased gradually after LCSE treatment in NCI-H661, but the level of cyclin D increased. The elevation of Kip1/p27 level in NCI-H661cells treated with dosage of LCSE greater than 25 μg/mL was also noted. These results indicated that LCSE arrested A549 cells in the G1 phase of the cell cycle and concomitantly inhibited G2/M progression, possibly by suppressing the expressions of cyclin D1, E and B1 and increasing the Kip1/p27 level. The suppression of cyclin with elevation of Kip1/p27 accounted for the LCSE arresting NCI-H661 cell in the G2/M phase of the cell cycle.

### Apoptosis induction by LCSE in NSCLC cells

**Fig. 3:**
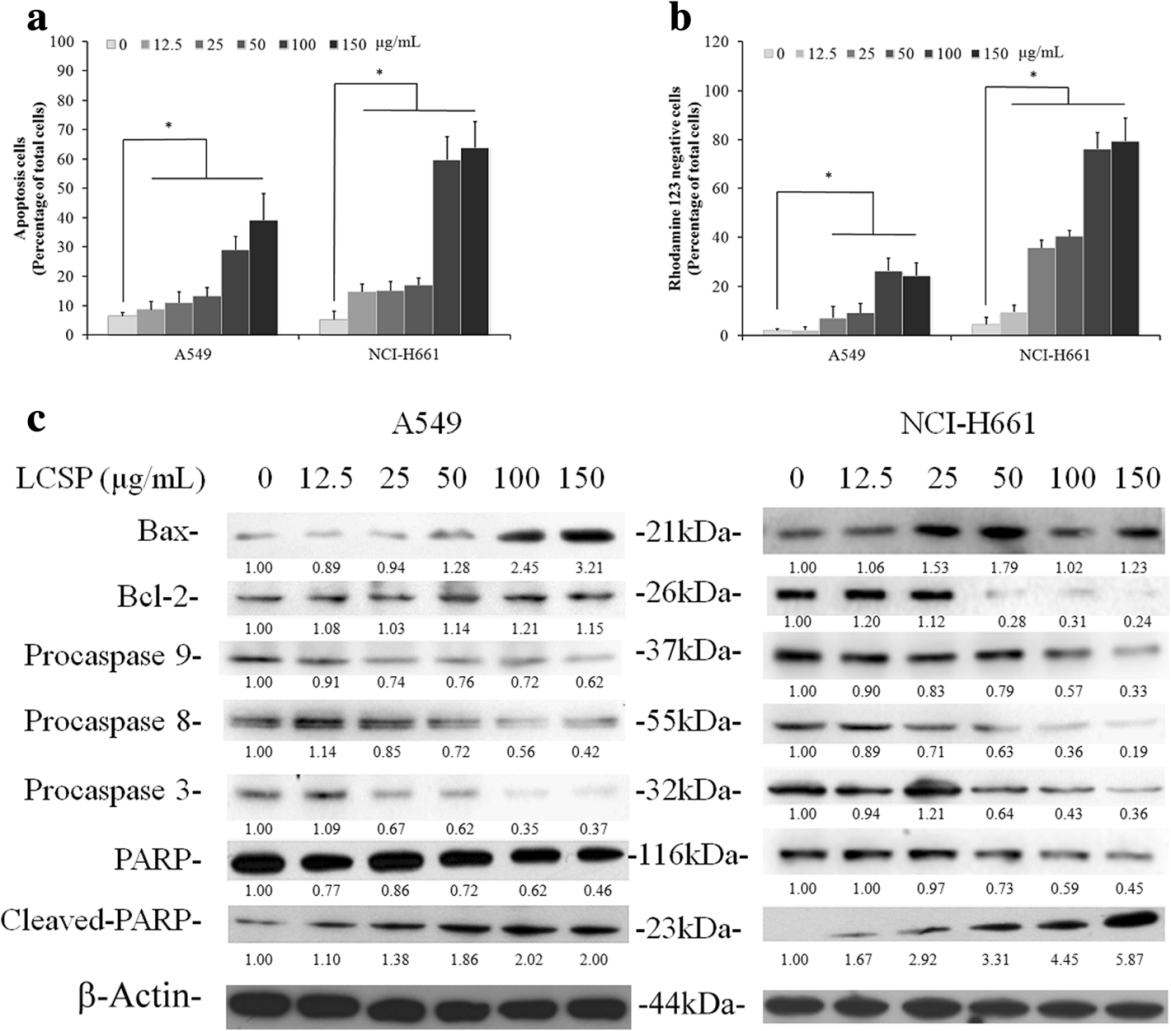
LCSE induced apoptosis in NSCLC cells. LCSE-treated cells were incubated at 37 °C for 24 h, stained with annexin V conjugated with FITC (**a**) or rhodamine 123 (**b**), then analyzed by flow cytometry, as described in Materials and Methods. Cell protein lysates from LCSE-treated cells were separated by SDS-PAGE, transferred to PVDF membranes, and immunoblotted to show proteins as indicated. Protein levels were quantified and normalized using the density of the beta-actin level as the loading control (**c**). The data reported are the averages of three independent experiments and are expressed as means ± SD. * *P* < 0.05

LCSE-treated A549 and NCI-H661 cells were stained with annexin V-FITC or rhodamine 123, and were analyzed by flow cytometry to assess the numbers of apoptotic cells and the mitochondrial integrity. Compared with the control groups, the number of annexin V-positive cells in both LCSE-treated NSCLC cell lines gradually increased according to the dosage elevation. A549 was shown to be less affected by LCSE-induced apoptosis. Contrary to A541, the increasing percentage of annexin V-positive cells from 12% in 25 μg/mL group to 40% in 150 μg/mL group of LCSE treatment, approximately 14% of annexin V-positive cells were observed in 12.5 μg/mL group, and increased to 64% in 150 μg/mLgroup of LCSE-treated NCI-H661 cells (Fig. 3a). To investigate whether the induction of apoptosis by LCSE in NSCLC cells involved the loss of mitochondrial integrity, *Δψ*_*m*_ in LCSE-treated cells was analyzed. As shown in Fig. 3b, the percentages of rhodamine 123-negative cells in the control groups were all below 5% of the total cells. After treatment with LCSE, this percentage increased significantly (*P* < 0.05), from 13% in the group of 12.5 μg/mL to around 80% in the 150 μg/mL group of LCSE-treated NCI-H661 cells. The percentage of rhodamine 123-negative A549 cells also increased from around10% in 25 μg/mL group to around 25% in the 150 μg/mL LCSE treatment. Immunoblotting analysis revealed that the levels of pro-caspase 8, 9 and 3 were decreased when treated with dosage greater than 25 μg/mL LCSE, while the caspase 3 substrate PARP showed increasing levels of cleaved fragments under the same treatment conditions in A549 cells (Fig. 3b). The level of apoptosis-promoting protein Bax was increased in A549 cells when treated with greater than 50 μg/mL LCSE. However, the level of anti-apoptosis protein Bcl-2 was sustained under LCSE treatment in A549 cells. The levels of pro-caspase 8, 9 and 3 were gradually decreased and the caspase 3 substrate PARP showed increasing levels of cleaved fragments in LCSE treated NCI-H661 cells that were similar to A549 cells with dosage greater than 12.5 μg/mL. The level of apoptosis-promoting protein Bax was increased in NCI-H661 cells under treatment with dosage greater than 25 μg/mL LCSE, while the level of anti-apoptosis protein Bcl-2 was decreased under treatment with dosage greater than 50 μg/mL LCSE in NCI-H661.

### LCSE disturbed EGFR signaling in NSCLC cells

**Fig. 4:**
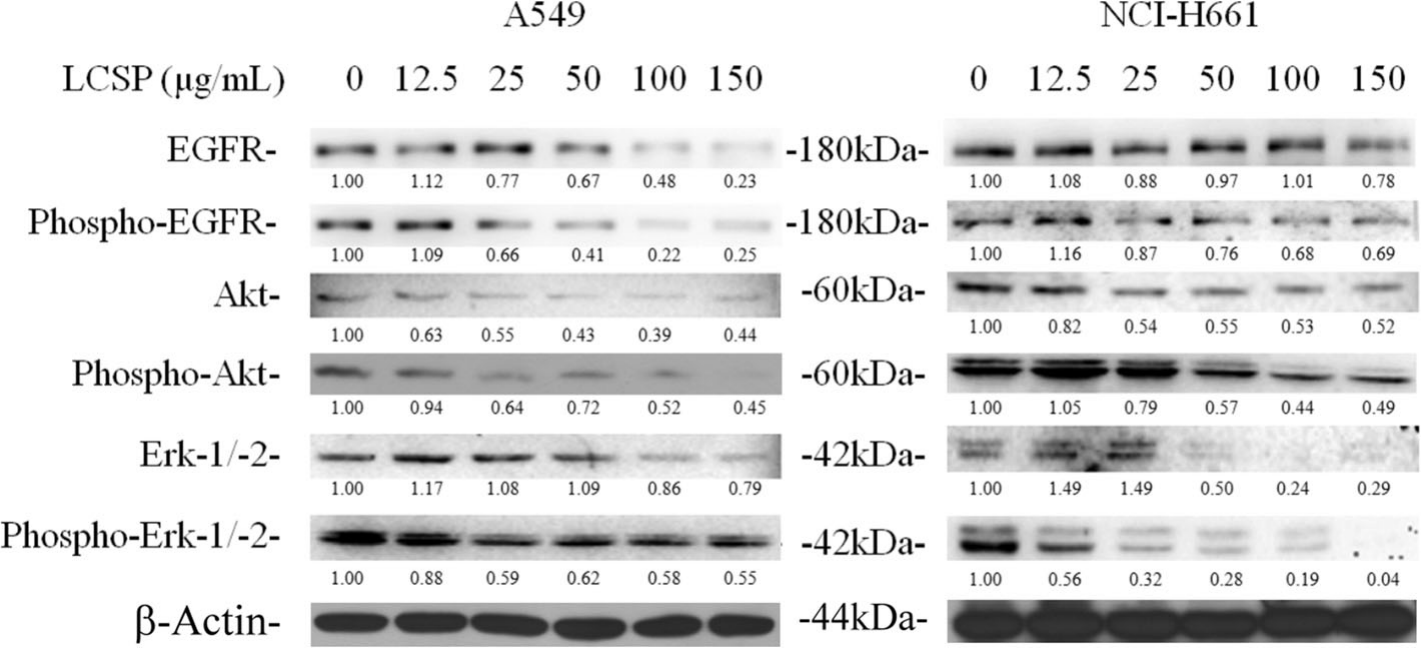
Immunoblots of EGFR signaling in LCSE-treated NSCLC cells. Cells were treated with increasing concentrations of LCSE as indicated and then incubated at 37 °C for 24 h. Cell protein lysates from A549 and NCI-H661 cells were separated by SDS-PAGE, transferred to PVDF membranes, and immunoblotted to show proteins and their phosphorylation levels, with the beta-actin level used as the loading control

EGFR protein and its phosphorylated tyrosine level were gradually decreased in A549 cells treated with dosage greater than 25 μg/mL LCSE (Fig. 4). The expression in the protein level of EGFR was unremarkable, while the phosphor-EGFR level was decreased in cells treated with dosage greater than 25 μg/mL of LCSE in NCI-H661 cells. Both the protein level and phosphorylation level of EGFR downstream signal protein Akt were decreased in the two LCSE-treated NSCLC cell lines. The protein levels of Erk-1 and −2 were not altered under LCSE treatment below 50 μg/mL but were slightly decreased under treatment with greater than 100 μg/mL of LCSE in A549 cells. The levels of phosphor-Erk-1 and −2 were decreased in cells treated with dosage greater than 25 μg/mL of LCSE in A549 cells. The levels of these two Erk proteins were slightly increased when treated with dosage below 25 μg/mL of LCSE and were decreased under the dosage greater than 50 μg/mL in NCI-H661. The levels of phosphor-Erk-1 and −2 were significantly decreased under treatment with dosage greater than 12.5 μg/mL LCSE and were undetectable under treatment with dosage of 150 μg/mL LCSE in NCI-H661 cells.

## Discussion

Previous studies have revealed inhibitory effects of LCSE on the growth of several types of malignant cells, including lung cancer [8, 11]. However, the exact mechanisms responsible for the anti-cancer activity of LCSE are still not fully understood. In this study, we comprehensively investigated the possible cellular and molecular mechanisms of the effects of LCSE in two NSCLC cancer cell lines, A549 and NCI-H661. The results demonstrated that LCSE suppressed the EGFR protein and its phosphorylation levels, then downregulated the downstream signaling, such as Erk-1/-2 and Akt signaling. These effects resulted in the suppression of some cyclins, elevation of the expressions of Kip1/p27 and Bax protein, and activation of caspase-8, −9 and −3, which induced cell-cycle arrest and apoptosis in NSCLC cells.

EGFR is the predominant target of therapy in NSCLC, as this proto-oncogene is always overexpressed and plays a key role in the growth of NSCLC [16]. Activated EGFR stimulates its two major downstream signaling pathways, Ras and phosphatidyl inositol 3 kinase (PI3K), then activates Erk-1/-2 and Akt signaling. Erk-1/-2 translocates to the nucleus and triggers expression of cell-cycle-controlling proteins. Akt could phosphorylate Bad and dissociate Bad with Bcl-2, which suppresses the activity of Bax and leads to the avoidance of apoptosis in cancer cells [10]. In this study, we demonstrated the elevation of the number of apoptotic cells, and the reduction in mitochondria membrane potential in both treated A549 and NCI-H661cells with LCSE. The expression of Bax protein was upregulated in both cells, and Bcl-2 was downregulated in NCI-H661 cells. These results indicated that the inhibitory effect of LCSE on EGFR signaling directly effected the Akt activity, then triggered an intrinsic apoptosis mechanism to induce apoptotic death in both NSCLC cells. A previous report indicated that LCSE also triggers the other extrinsic apoptosis pathway to induce apoptosis [10]. Our results appeared to support this viewpoint, with the level of procaspase 8 being gradually decreased after LCSE treatment in the NSCLC cells. From our results and those of other studies, we can conclude that LCSE-induced apoptotic death in NSCLC cells arises from triggering both the intrinsic and extrinsic apoptosis pathways.

Although LCSE induced apoptosis in both tested NSCLC cells, the sensitivity differed between the two cells. NCI-H661 cells were more sensitive than A549 cells in the treatment with LCSE which was demonstrated by the number of annexin V-positive cells, the mitochondria potential loss and the Bax and Bcl-2 protein levels. As shown in Fig. 3, the number of annexin V-positive cells and the mitochondria membrane potential loss of cells were elevated more than 60% of the total NCI-H661 cells under LCSE treatment with dosage of 100 and 150 μg/mL. In the A549 cells, only less than 40% of the total number of cells underwent apoptosis. Although the Bax level in LCSE-treated cells was elevated, the Bcl-2 level in NCI-H661 cells was dramatically diminished under dosage more than 50 μg/mL LCSE treatment, whereas the level of Bcl-2 in A549 cells was sustained. The balance of Bax and Bcl-2 in cells plays a pivotal role in the regulation of apoptosis in LCSE-treated cancer cells which was highlighted in our previous report and other studies [8, 11, 12]. LCSE-treated NCI-H661 cells contained a greater number of apoptotic cells indicating that the mechanism of the growth inhibition effect of LCSE in the cells is mitochondria-mediated apoptosis, which mainly arised from elevation of the Bax level and downregulation of the Bcl-2 level.

Our previous study revealed that LCSE arrested cancer cells in the G2/M phase by reducing the expression of cyclin B1 [12]. In this study, we further confirmed this effect in the two tested NSCLC cells. The number of NCI-H661 cells in the G2/M phase was elevated under treatment with LCSE with dosage more than 12.5 μg/mL. However, the change of G2/M phase only gradually increased to a quarter of A549 cells under LCSE treament with dosage of 150 μg/mL. These results suggest the effect of cell cycle arrest in G2/M phase was more predominant in NCI-H661 cells. Previous studies revealed that Erk activity is an important factor in G2/M-phase progression in the cell cycle, and the M phase is nearly twice as long in mammalian cultured cells when Erk is inhibited during G2/M transition [17, 18]. In the report of Lin et al., they indicated that downregulation of the EGFR/Erk/c-fos signaling pathway can inhibit the COX-2 level and activate Kip1/p27 to induce G2/M cell-cycle arrest in oral carcinoma cells [19]. Another study revealed that EGF could activate Akt and triggered CK2a and HDAC2 expression; whereas inhibition of Akt or CK2a and concomitant suppression of the expressions of CDC25c and cyclin B would arrest cells in the G2/M phase [20]. Our results proved that LCSE inhibited the activities of EGFR, Erk-1/-2 and Akt and concomitantly suppressed the level of cyclin B; therefore, indicating that the LCSE-induced G2/M-phase arrest might arise from the reduction of EGFR signaling and control of cyclin B in NSCLC cells.

However, LCSE also disturbed the process of the G1 phase progression in A549 cells, including suppression of the levels of cyclin D1 and E and elevation of the level of Kip1/p27. Our results confirmed that LCSE-treated A549 cells expressed a greater number of G1-phase cells as compared to control cells. Recent studies proved that suppression of the EGFR/Ras/MEK/Erk and PI-3 K/Akt pathways could lead to cell-cycle arrest in the G1 phase [21, 22], mainly through upregulation of Kip/p27 and downregulation of cyclin E [23, 24]. Earlier reports also pointed out that Erk regulates cyclin D1 transcription and cyclin D1/CDKs assembly and triggers G1 to S transition in the cell cycle [25–29]. Since LCSE reduced EGFR signaling, downregulated Erk-1/-2 and Akt, and arrested the G1 phase in A549 cells, our results indicated a possible novel role of LCSE in the control of lung adenocarcinoma cells in terms of cell-cycle progression from the G1 phase to the S phase. However, the effect of G1 phase arrest was not fully apparent in LCSE-treated NCI-H661 cells, in which cyclin D1 was elevated under LCSE treatment. The reasons for these results need further investigation.

## Conclusion

Our results revealed that LCSE inhibited EGFR activity and protein expression in two NSCLC cells, concomitantly reduced the downstream Akt and Erk-1/-2 signaling, then downregulated Bcl-2, cyclin B1 and cyclin E and elevated Kip1/p27 and Bax. In addition to growth inhibition in both cell lines, these events resulted in cell arrest at the G2/M phase of the cell cycle and lead to apoptotic death. This study was the first to describe the detailed mechanisms of LCSE in NSCLC and suggested LCSE may be a novel herbal agent that acts through the inhibition of EGFR signaling to induce cytotoxicity in NSCLC. It would be intriguing to examine whether the findings of this study could be replicated in an animal model of NSCLC and further investigation is warranted.

## Abbreviations

BCA: Bicinchoninic acid
FITC: Fluorescein isothiocyanate
LCSE: Litchi seed extract
NSCLC: Non-small cell lung cancer
PAGE: Polyacrylamide gel electrophoresis
PARP: Poly[ADP-ribose] polymerase
PVDF: Polyvinylidene fluoride
SDS: Sodium dodecyl sulfate
TKI: Tyrosine kinase inhibitor
*Δφ*_*m*_: Mitochondria membrane potential

## References

[1] Ellis PM, Coakley N, Feld R, Kuruvilla S, Ung YC (2015). Use of the epidermal growth factor receptor inhibitors gefitinib, erlotinib, afatinib, dacomitinib, and icotinib in the treatment of non-small-cell lung cancer: a systematic review. Curr Oncol.

[2] Sculier JP, Berghmans T, Meert AP (2015). Advances in target therapy in lung cancer. Eur Respir Rev.

[3] West H, Oxnard GR, Doebele RC. Acquired resistance to targeted therapies in advanced non-small cell lung cancer: new strategies and new agents. Am Soc Clin Oncol Educ Book. 2013. doi: 10.1200/EdBook_AM.2013.33.e272. 10.1200/EdBook_AM.2013.33.e272 PMC4142045 23714521

[4] Linardou H, Dahabreh IJ, Kanaloupiti D, Siannis F, Bafaloukos D, Kosmidis P, Papadimitriou CA, Murray S (2008). Assessment of somatic k-RAS mutations as a mechanism associated with resistance to EGFR-targeted agents: a systematic review and meta-analysis of studies in advanced non-small-cell lung cancer and metastatic colorectal cancer. Lancet Oncol.

[5] Gontiner E, Boussouel N, Terrasse C, Jannoyer M, Ménard M, Thomasset B, Bourgaud F (2000). Litchi Chinensis fatty acid diversity: occurrence of the unusual cyclopropanoic fatty acids. Biochem Soc Trans.

[6] Li J, Jiang Y (2007). Litchi flavnoids: isolation, identification and biological activity. Molecules.

[7] Huang F, Zhang R, Yang Y (2014). Comparison of physicochemical properties and immunomodulatory activity of polysaccharides from fresh and dried litchi pulp. Molecules.

[8] Xu X, Xie H, Hao J, Jiang Y, Wei X (2011). Flavnoid glycosides from the seeds of Litchi Chinensis. J Agric Food Chem.

[9] Xu X, Xie H, Wang Y, Wei X (2010). A-type proanthocyanidins from lychee seeds and their antioxidant and antiviral activities. J Agric Food Chem.

[10] Zhang JY, Zhang C (2015). Research progress on the antineoplastic pharmacological effects and mechanisms of Litchi seeds. Chin Med.

[11] Lin CC, Chung YC, Hsu CP (2013). Anti-cancer potential of Litchi seed extract. World J Exp Med.

[12] Hsu CP, Lin CC, Huang CC, Lin YH, Chou JC, Tsia YT, Su JR, Chung YC (2012). Induction of apoptosis and cell cycle arrest in human colorectal carcinoma by Litchi seed extract. J Biomed Biotechnol.

[13] Chung YC, Lin CC, Chou CC, Hsu CP (2010). The effect of longan seed polyphenols on colorectal carcinoma cells. Euro J Clin Invest.

[14] Chung YC, Huang CC, Chen CH, Chiang HC, Chen KB, Chen YJ, Liu CL, Chuang LT, Liu M, Hsu CP (2012). Grape-seed procyanidins inhibit the in vitro growth and invasion of pancreatic carcinoma cells. Pancreas.

[15] Hsu CP, Lin YH, Zhou SP, Chung YC, Lin CC, Wang SC (2010). Longan flower extract inhibits the growth of colorectal carcinoma. Nutri Cancer.

[16] Iida M, Brand TM, Campbell DA, Starr MM, Luthar N, Traynor AM, Wheeler DL (2013). Targeting AKT with the allosteric AKT inhibitor MK-2206 in non-small cell lung cancer cells with acquired resistance to cetuximab. Cancer Biol Ther.

[17] Roberts EC, Shapiro PS, Nahreini TS, Pages G, Pouyssegur J, Ahn NG (2002). Distinct cell cycle timing requirements for extracellular signal-regulated kinase and phosphoinositide 3-kinase signaling pathways in somatic cell mitosis. Mol Cell Biol.

[18] Wright JH, Munar E, Jameson DR, Andreassen PR, Margolis RL, Seger R, Krebs EG (1999). Mitogen-activated protein kinase kinase activity is required for the G(2)/M transition of the cell cycle in mammalian fibroblasts. Proc Natl Acad Sci U S A.

[19] Lin YM, Kuo WW, Velmurugan BK, Hsien HH, Hsieh YL, Hsu HH, Tu CC, Bau DT, Viswanadha VP, Huang CY. Helioxanthin suppresses the cross talk of COX-2/PGE2 and EGFR/ERK pathway to inhibit Arecoline-induced Oral Cancer Cell (T28) proliferation and blocks tumor growth in xenografted nude mice. Environ Toxicol. 2015; doi: 10.1002/tox.22204. 10.1002/tox.22204 26464283

[20] Kim HS, Chang YG, Bae HJ, Eun JW, Shen Q, Park SJ, Shin WC, Lee EK, Park S, Ahn YM, Park WS, Lee JY, Nam SW (2014). Oncogenic potential of CK2α and its regulatory role in EGF-induced HDAC2 expression in human liver cancer. FEBS J.

[21] Okumura S, Jänne PA (2014). Molecular pathways: the basis for rational combination using MEK inhibitors in KRAS-mutant cancers. Clin Cancer Res.

[22] Moreno-Layseca P, Streuli CH (2014). Signalling pathways linking integrins with cell cycle progression. Matrix Biol.

[23] Kirsammer G, Strizzi L, Margaryan NV, Gilgur A, Hyser M, Atkinson J, Kirschmann DA, Seftor EA, Hendrix MJ (2014). Nodal signaling promotes a tumorigenic phenotype in human breast cancer. Semin Cancer Biol.

[24] Hlobilková A, Knillová J, Bártek J, Lukás J, Kolár Z (2003). The mechanism of action of the tumour suppressor gene PTEN. Biomed Pap Med Fac Univ Palacky Olomouc Czech Repub.

[25] Chambard JC, Lefloch R, Pouysségur J, Lenormand P (2007). ERK implication in cell cycle regulation. Biochim Biophys Acta.

[26] Keenan SM, Bellone C, Baldassare JJ (2001). Cyclin-dependent kinase 2 nucleocytoplasmic translocation is regulated by extracellular regulated kinase. J Biol Chem.

[27] Sherr CJ, Roberts JM (1999). CDK inhibitors: positive and negative regulators of G1-phase progression. Genes Dev.

[28] Weber JD, Raben DM, Phillips PJ, Baldassare JJ (1997). Sustained activation of extracellular-signal-regulated kinase 1 (ERK1) is required for the continued expression of cyclin D1 in G1 phase. Biochem J.

[29] Lavoie JN, L’Allemain G, Brunet A, Müller R, Pouysségur J (1996). Cyclin D1 expression is regulated positively by the p42/p44MAPK and negatively by the p38/HOGMAPK pathway. J Biol Chem.

